# Protein translocation: what's the problem?

**DOI:** 10.1042/BST20160047

**Published:** 2016-06-09

**Authors:** Robin A. Corey, William J. Allen, Ian Collinson

**Affiliations:** * School of Biochemistry, University of Bristol, University Walk, BS8 1TD, U.K.

**Keywords:** ATPase, energy transduction, membrane protein complex, protein translocation, secretion, SecY

## Abstract

We came together in Leeds to commemorate and celebrate the life and achievements of Prof. Stephen Baldwin. For many years we, together with Sheena Radford and Roman Tuma (colleagues also of the University of Leeds), have worked together on the problem of protein translocation through the essential and ubiquitous Sec system. Inspired and helped by Steve we may finally be making progress. My seminar described our latest hypothesis for the molecular mechanism of protein translocation, supported by results collected in Bristol and Leeds on the tractable bacterial secretion process–commonly known as the Sec system; work that will be published elsewhere. Below is a description of the alternative and contested models for protein translocation that we all have been contemplating for many years. This review will consider their *pros* and *cons*.

Between 20% and 30% of all proteins are translocated across or inserted into lipid membranes [1,2]. The challenge of transporting a long and charged polymer with varying hydrophobicity and hydrophilicity across or into a semi-impermeable barrier is indeed great. It has to be done specifically, so that only the right proteins end up the right place, and it has to be done without compromising the barrier posed by the membrane–necessary for compartmentalization and energy conservation. These problems are overcome by specialized membrane protein complexes called translocons.

## The Sec pathway

The Sec machinery is found in every cell in every organism, wherein translocation occurs through a hetero-trimeric membrane protein core complex: the SecY-complex in bacteria [3], archaea [4] and chloroplast thylakoid membranes [5] and Sec61 in eukaryotes [6]. Translocation through the Sec protein channel occurs either co-translationally, by engaging translating ribosomes, or post-translationally. The post-translational pathway is the main pathway for protein secretion in prokaryotes [7], acting on unfolded pre-proteins [8,9]. The co-translational pathway is used by eukaryotes for secretion [10], and across most species for membrane protein insertion [11].

For both pathways, translocation is initiated upon targeting of a transport substrate to the SecY/Sec61 complex at the plasma/ER membrane, via a cleavable N-terminal signal sequence (SS) for secretory proteins or the first TM of nascent membrane proteins (the signal anchor; SA). For the co-translational pathway, this involves the delivery of the ribosome nascent chain (RNC) complex to the membrane, guided by the signal recognition particle and its cognate receptor [12]; the subsequently formed Sec–RNC complex has been described structurally at medium resolution by electron cryo-microscopy [13–16]. In bacteria, the post translational process of protein secretion is assisted by the auxiliary proteins SecD, SecF, YajC [17,18]; whereas co-translational membrane protein insertion is facilitated by YidC [19,20], with the probable combination of all of these factors into a ‘holo-translocon’ capable of both secretion and insertion [21].

**Figure 1:**
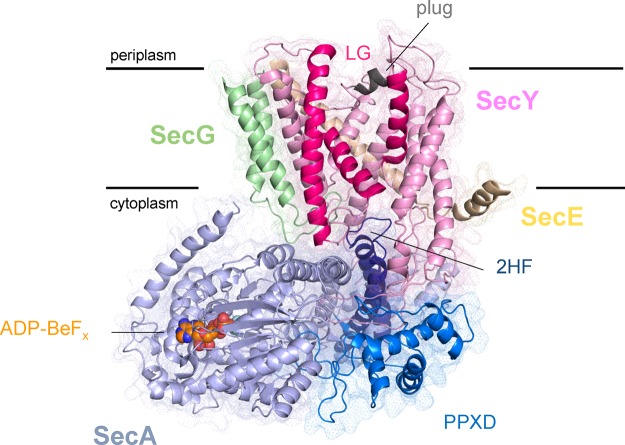
Structure of SecYEG–SecA *T. maritima* SecYEG–SecA (PDB code 3DIN [29]). Proteins are represented as cartoons with mesh surfaces. SecY is light pink, with the partly opened LG helices highlighted in dark pink and the plug as grey. SecE is shown in orange, SecG in green and SecA in light-blue, with the 2HF and PPXD coloured separately. The ATP analogue (ADP-BeF_x_) is coloured as orange, blue and red spheres. The approximate position of the membrane is indicated.

During the post-translational targeting process in bacteria, pre-secretory proteins with a cleavable N-terminal SS often engage a chaperone; for instance *E. coli* employ SecB *inter alia* [22]. The role of the chaperone is primarily to maintain the substrate in an unfolded conformation, in preparation for threading through the SecY channel. Next, the chaperone and pre-protein are jointly targeted to the dimeric SecA motor ATPase for post-translational transport through the membrane-bound SecYEG [23], whereupon the dimers of SecA dissociate [24]. It should be noted that SecA has been shown to interact directly with the ribosome and the exit tunnel [25], thus probably mitigating the need for chaperones in certain cases. Irrespective of the route taken, once SecA and the pre-protein are engaged with SecYEG, the channel is ‘unlocked’ by the SS [26–28] and ‘activated’ by the binding of monomeric SecA [29] (Figure 1) prior to polypeptide intercalation and transport.

## Structure of the SecYEG complex

SecYEG has three membrane protein subunits, with the largest and most important being SecY. A crystal structure of *Methanococcus jannaschii* SecYEβ at 3.2 Å [30] reveals SecY to have 10 TMs arranged in a claw-like structure, formed by the N- and C-terminal membrane domains (TMs 1–5 and 6–10). These two halves form an hourglass shaped trans-membrane pore through which protein translocation occurs [31]. The pore is closed in the centre by a ring of six hydrophobic residues and a helical plug, which may embrace the translocating chain to prevent the undesirable flow of small molecules and ions through the channel during the transport process [30,32]. A lateral gate (LG) is formed where the two domains of SecY meet, between TMs 2/3 and 7/8, for the passage of trans-membrane helices into the bilayer. The LG is also the site of secretory SS binding [13,26].

## The SecA motor ATPase

SecA is a superfamily 2 RNA helicase, which converts the chemical energy from ATP into directional protein translocation through SecYEG [33]. In addition to the nucleotide binding domains (NBD1 and NBD2), between which ATP is bound and hydrolysed, there are additional domains, which are apparently crucial for the recognition, binding and translocation of secretory pre-proteins. Most important of these are the peptide cross-linking domain (PPXD) and the so-called two-helix finger (2HF) [34], both of which contact the translocating polypeptide [35]. A key role for the 2HF in protein translocation has previously been noted [36,37], with its positioning at the SecA–SecY interface making it a likely contender for both pre-protein [35] and SecY interaction [38].

## Binding and activation of SecA and SecYEG

In the bacterial system, SecA binds SecYEG to initiate post-translational protein translocation [39]. The comparison of structural data for SecYEG [30] and SecA [34] in their resting states with the *Thermotoga maritima* SecYEG–SecA complex–bound to a non-hydrolysable analogue of ATP–reveals numerous conformational changes in both SecA and SecY (Figure 1) [29]. In SecA, the PPXD moves towards the NBD2 by about 25 Å, forming a clamp that prevents dissociation of the translocating pre-protein [29]. The 2HF also rotates, protruding into the SecY channel. These conformational changes are accompanied by stimulation of the ATPase activity in SecA, from its very slow basal level [40]. For SecY, the principal rearrangements manifest in the cytoplasmic loops, which rearrange to mediate tight binding to SecA. These movements are accompanied by a partial opening of the channel and a widening of the LG, which in turn perturbs the plug [29,41]–perhaps facilitating its displacement by an incoming pre-protein.

**Figure 2:**
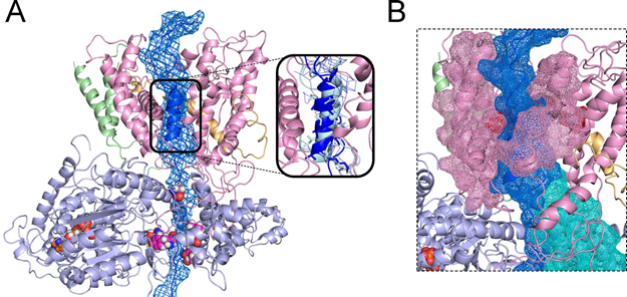
Modelling a pre-protein path through the complex (**A**) Model built based on the SecYEG–SecA crystal structure (PDB code 3DIN [29]) which has been allowed to relax with MD simulations (full details to be published separately). The proteins are shown as cartoons, with SecA in light blue, SecY in pink, SecE in light orange and SecG in light green, with a bound ATP molecule shown as orange, blue, white and red spheres. A prospective pathway for a model substrate (the first 76 residues of pro-OmpA; shown as dark blue cartoon and mesh) has been built into the channel based on known cross-linking sites in SecA (pink, red, blue and white spheres; [35]) and the position of the SecY pore ring [30]. The helical SS was built based on the density of the DsbA SS from a recent cryo-EM structure of the SecY complex bound to a ribosome (inset–DsbA SS light blue, with the density shown using map EMD-5693, at 3.0 sigma within 2.6 Å of the selection [13]). (**B**) Close-up of the channel from (A) with the substrate, LG and 2HF shown as dark blue, pink and teal mesh respectively. The pore ring residues of SecY are highlighted in red.

The widening of the LG opens up a gap between the hydrophilic channel and the hydrophobic membrane interior, with sufficient width to accommodate the α-helical SS. Indeed, structural data has routinely sited the SS in this region [14,15,26,41], with further support lent by cross-linking studies [42–44]. Although no structure yet exists of the entire SecYEG–SecA–substrate complex, enough evidence is available to localize the various components: we have combined the structurally determined position of the SS [13,26] with the known route of the substrate through SecY [30,31] and SecA [35] to produce a plausible model of pre-protein positioning within the complex during translocation (Figure 2A). Although speculative, the model represents a likely approximate pathway for the pre-protein, and demonstrates the tightness of space within the channel (Figure 2B).

The association of SS with the LG acts as an allosteric activator of the SecYEG complex, ‘unlocking’ the channel and priming it for transport of the rest of the pre-protein [26–28,41]. The membrane exposure of the SS binding site provides a proofreading step: sequences that are too hydrophilic are excluded, and presumably fail to unlock the channel for productive transport. During the co-translational process of membrane protein insertion, which is independent of SecA, hydrophobic transmembrane helices partition from this location laterally into the bilayer [23].

## Current models of Sec-mediated protein translocation in prokaryotes

The secretion process can in essence be described as two distinct steps: activation/initiation (outlined above) and translocation. There is enough structural detail available to mock up the post-initiation state (Figure 2A), the assembly of which involves the dissociation of SecA dimers [24], the relocation of the PPXD [29] (described above) and the activation of the ATPase, as proposed previously [40]. Less is understood about the bioenergetics of the transport mechanism, i.e. how ATP hydrolysis and the trans-membrane proton motive force (PMF) cooperate to push the rest of the polypeptide across the membrane.

**Figure 3:**
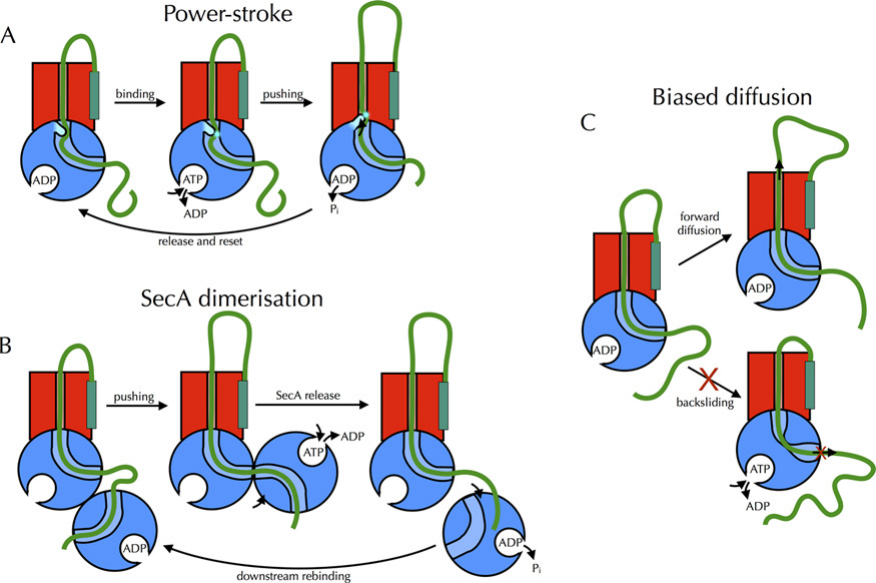
Previously proposed models for translocation by the Sec complex Many previous models for how SecA drives translocation have been proposed, designed to accommodate the results of structural and functional studies. So far, however, such models make various assumptions, e.g. they postulate the existence of conformational changes that lack direct experimental evidence. In general these can be divided into three types: models involving a power stroke within SecA, those that invoke quaternary interactions between multiple SecA molecules or those which act through biased diffusion. (**A**) An example power-stroke mechanism, whereby conformational changes within SecA during the ATPase cycle physically push polypeptides through the channel. In the model shown [36], the 2HF binds to the pre-protein substrate, pushes it into the channel, then releases it and returns to its resting position. (**B**) The observation that SecA can exist both as a monomer and in several different dimer forms has led to the proposal of multiple models in which quaternary interactions drive transport. In the example shown, one SecA protomer holds the pre-protein substrate in the channel whereas the other binds to downstream regions. ATP binding alters the SecA dimer interface, pushing the substrate through the channel, whereas ATP hydrolysis releases SecA, allowing it to rebind downstream. (**C**) Rather than physically pushing the substrate through the channel, directional movement can be achieved by selectively allowing diffusion in one direction, while preventing it in the other. Such a ‘Brownian ratchet’ would act by using ATP to somehow prevent backsliding. In the version shown, SecA senses backsliding and constricts to halt movement; however this is entirely speculative, as an illustration of the core concept.

In contrast with the sequence-dependent (SS recognition) initiation process, the subsequent translocation of the polypeptide is far less specific. A considerable variety of proteins are transported through SecY, and these inevitably contain a range of different sequences, including stretches of hydrophobic and charged amino acids; it is not easy to envisage a process that will recognize and transport them all. Nonetheless, several mechanisms have been suggested, with varying levels of experimental support. These can be broadly divided into three categories: those driven by a power-stroke within SecA; those that involve quaternary interactions between multiple SecA molecules and those that bias the direction of diffusion across the membrane (Figure 3). However, these are not necessarily mutually exclusive, and models proposed more recently tend to contain combinations of all three–perhaps in an attempt to rationalize the mass of apparently conflicting data accumulated over the past 25 years.

## Power-stroke models

Power-stroke models invoke the physical pushing of pre-protein through the channel and across the membrane, driven by multiple rounds of ATP hydrolysis (Figure 3A) [45]. Each ATP turnover cycle transports a short stretch of peptide, then releases it and resets to bind upstream [36,37,46]. Such mechanisms are compelling in one respect, as they mimic the DEAD-box helicases, to which SecA is related [34]–although secretory pre-proteins lack the uniformity of the phosphate backbone. However, as we have argued previously [47], the major observations that led to the original proposal of the power-stroke model–particularly the intermediate translocation products that can sometimes be seen at low ATP concentrations [48,49]–are by no means conclusive, and are very much open to interpretation.

A critical component of a power stroke would be an ATP-dependent conformational change that could push the peptide. However, none of those suggested conformational changes appear to be necessary for its function. At present, the 2HF is the most plausible candidate for the role of piston: it sits directly on the path of the pre-protein substrate as it enters the SecY channel [35], and has been proposed to alternately enter and retract from the channel, pushing substrate in one direction [36].

A number of objections can be raised to the 2HF-power-stroke model. Firstly, there is little evidence that the 2HF can retract from the channel when SecA is bound to SecY. Indeed, the interface between SecY and SecA appears to be a very snug fit, with very little wiggle room (Figure 2B). We have also shown that the complex remains functional even when the 2HF is cross-linked by a disulfide bond into SecY [38]–any movement would therefore have to be very subtle indeed, rendering any polypeptide pushing capabilities ineffectual. A more general concern for all power-stroke models is the broad variety of possible sequences that must be transported: how could one single binding site push the hydrophobic core of a β-barrel outer membrane protein in one stroke, then its positively or negatively charged periplasmic loop in the next?

## SecA dimerization models

The oligomeric state of SecA is a controversial topic within the field. It is a dimer when free in solution [50–52], but upon association with SecY it either monomerizes [24,53] or forms one of a number of different dimers, seemingly dependent on the experimental condition (see e.g. [27]). This has led to the suggestion that quaternary interactions between multiple SecA protomers might drive translocation: perhaps ATP-dependent rearrangements in the SecA dimer interface [54,55]–or indeed alternate monomerization and dimerization [56]–push the substrate through the channel (Figure 3B)?

More complicated models have been also proposed, which combine power strokes and monomer–dimer transitions. For example, the ‘reciprocating piston’ requires SecA to undergo some quite startling gymnastics in order to achieve pre-protein delivery across the membrane [46]. It has even been proposed that different substrates require different stoichiometries of SecYEG:SecA [57]. Although the model Mao et al. propose uses SecB, and so cannot be universal (not all bacteria have SecB) it should certainly be borne in mind that most studies are carried out using a small number of model translocation substrates. It may well be that different mechanisms are used depending on the substrate being transported.

## Diffusional ratchet

The actual nuts and bolts of protein secretion are perhaps better understood for eukaryotic systems compared with bacteria. In yeast, the bound pre-protein is able to freely diffuse back and forth through the Sec61 channel by random Brownian motion. As the polypeptide passes into the ER lumen, it is recognized and bound by the Hsp70 homologue BiP [58], in an ATP-dependent manner. This binding prevents the diffusion of the pre-protein back through the channel, thus biases the direction of diffusion–and hence translocation–in a forward direction. Such a mechanism, which functions by converting random thermal energy into directional motion, can be referred to as a Brownian ratchet (Figure 3C).

This is similar to possible diffusional ratchet mechanisms of protein secretion in bacteria, whereby turnover of ATP is coupled to a ratcheting of the pre-protein, acting to bias the direction of diffusion through SecYEG. The primary difference is that, as bacteria do not have ATP on the exterior side of their membrane, secretion must be powered from the cytoplasmic side. Alternatively, the ratcheting effect could arise from an as-yet unknown chemical asymmetry across the membrane [59].

Models whereby protein translocation is powered by Brownian motion have many advantages, most prominently perhaps being in the speed of thermal motion at physiologically relevant temperatures [60]. Indeed, each copy of SecYEG probably secretes a pre-protein every second [47], in this regard making stochastic diffusion-based models more plausible than a processive step-wise mechanism.

In addition, the harnessing of random diffusion would require far less sequence specificity within the substrate pre-protein, providing that the channel can open enough to prevent strong interactions with the substrate. Furthermore, it should be relatively easy to speculate as to how the PMF cooperates with this process to stimulate the passage of pre-protein across the membrane, making extension of a model to incorporate PMF stimulation a distinct possibility.

## Concluding remarks

The protein translocation systems found in mitochondria, chloroplasts and the general secretory pathways (Sec and Tat) are responsible for the efficient delivery and folding of globular and membrane proteins into their correct compartment or into the membrane. They are all highly complex multi-subunit membrane-bound machines, the understanding of which is complicated–in the energy conserving membranes of bacteria, mitochondria and chloroplasts–by the use, in addition to ATP, of the PMF as an energy source. In spite of a generation of research since their discovery in the late 80s and 90s, the dynamic molecular mechanisms underlying transport have yet to be described. Of all these translocation systems, we understand most about the Sec machinery. This is largely due to the availability of high-resolution structures of the SecY complex, determined more than a decade ago in a resting state [30] and a few years later bound to the SecA motor ATPase [29]. In addition, there has been a recent flurry of structures determined by electron cryo-microscopy of the Sec complex engaged with the SS and nascent translocation substrates [13,14,41]. However, these structural snapshots do not really address the dynamic mechanism of protein translocation. Nevertheless, they provide the necessary framework for the determination of the dynamics of the system through a range of powerful ensemble and single molecule biophysical strategies. Only time (and further analyses) will reveal the nature of the transport process at play. The mechanism may be one of the possibilities described above, a hybrid of several of them, or even an unexpected mechanism, yet to be revealed. So watch this space!

## Abbreviations

2HF: two-helix finger
LG: lateral gate
NBD: nucleotide binding domain
PMF: proton motive force
RNC: ribosome nascent chain
SS: signal sequence
